# Increased autonomy with capacity-based mental health legislation in Norway: a qualitative study of patient experiences of having come off a community treatment order

**DOI:** 10.1186/s12913-022-07892-9

**Published:** 2022-04-07

**Authors:** Nina Camilla Wergeland, Åshild Fause, Astrid Karine Weber, Anett Beatrix Osnes Fause, Henriette Riley

**Affiliations:** 1 Division of Mental Health and Substance Abuse, University Hospital of North Norway and UiT, The Arctic University of Norway, Norway Tromsø,; 2 Department of Health and Care Sciences, Faculty of Health Sciences, UiT The Arctic University of Norway, Tromsø, Norway; 3 Division of Mental Health and Substance Abuse, University Hospital of North Norway, Tromsø, Norway; 4 Elden Law Firm, Tromsø, Norway

**Keywords:** Coercion, Community treatment order, Outpatient commitment, Capacity to consent, The Mental Health Care Act, Patient experiences, Self-determination, Autonomy

## Abstract

**Background:**

Capacity-based mental health legislation was introduced in Norway on 1 September 2017. The aim was to increase the autonomy of patients with severe mental illness and to bring mental health care in line with human rights.

The aim of this study is to explore patient experiences of how far the new legislation has enabled them to be involved in decisions on their treatment after they were assessed as capable of giving consent and had their community treatment order (CTO) revoked due to the change in the legislation.

**Method:**

Individual in-depth interviews were conducted from September 2019 to March 2020 with twelve people with experience as CTO patients. Interviews were transcribed and analysed using thematic analysis inspired by hermeneutics.

**Results:**

Almost all interviewees were receiving the same health care over two years after their CTO was terminated. Following the new legislation, they found it easier to be involved in treatment decisions when off a CTO than they had done in periods without a CTO before the amendment. Being assessed as having capacity to consent had enhanced their autonomy, their dialogues and their feeling of being respected in encounters with health care personnel. However, several participants felt insecure in such encounters and some still felt passive and lacking in initiative due to their previous experiences of coercion. They were worried about becoming acutely ill and again being subjected to involuntary treatment.

**Conclusion:**

The introduction of capacity-based mental health legislation seems to have fulfilled the intention that treatment and care should, as far as possible, be provided in accordance with patients’ wishes. Systematic assessment of capacity to consent seems to increase the focus on patients’ condition, level of functioning and opinions in care and treatment. Stricter requirements for health care providers to find solutions in cooperation with patients seem to lead to new forms of collaboration between patients and health care personnel, where patients have become more active participants in their own treatment and receive help to make more informed choices.

## Background

There is growing awareness of mental health patients’ right to self-determination. The Convention on the Rights of Persons with Disabilities (CRPD) was adopted by the UN General Assembly in 2006 and implemented in 2008 [1]. The CRPD aims to ensure that people with disabilities, such as severe mental illness, have the same basic human rights as other people. In an ongoing international debate, it is argued that mental health care legislation without conditions for the lack of capacity to consent to the use of coercion is discriminatory, because without such conditions it is assumed that patients with severe mental illness lack the capacity to consent [2, 3].

In Norway, there have been efforts for several decades to enhance the freedom and autonomy of mental health patients [4, 5]. In 2013 the CRPD was ratified in Norway [6] and in 2014 two amendments to the Norwegian constitution were adopted that protect the integrity and privacy of individuals [7]. The lack of any reduction in the use of coercion, as well as strong pressure from service user organizations, led to an amendment to the legislation in 2017 where lack of capacity to consent was introduced as an independent condition for the use of coercion under the Mental Health Care Act [8]. This change is an adaptation to the principles of the CRPD, and is intended to strengthen patients’ right to self-determination and legal protection [9]. The change to capacity-based legislation is also aimed at decreasing the importance of a patient’s diagnosis. Patients should be able to refuse treatment they do not want, or end treatment they have started, provided that they are capable of weighing up alternatives and understanding the consequences of their choices. Patients are still entitled to health care, and must be allowed to choose between different suitable forms of treatment. Only patients who are assessed to represent a danger to their own life, or the health or life of others are exempt from the condition of lack of capacity to consent [8]. The patient’s capacity to consent is assessed by the responsible psychiatrist or specialist clinical psychologist.

When introducing the change in the legislation, the government focused on these four areas in assessing patients’ capacity to consent: 1) the ability to understand information relevant to health care decisions, 2) the ability to apply the information to their own situation, especially in relation to their particular mental health problems and possible consequences of different treatment options, 3) the ability to use relevant information to weigh up treatment options, and 4) the ability to express a choice [10]. If there is any doubt as to whether the patient understands what consent entails, the patient must be allowed to refuse recommended treatment, while still being entitled to necessary health care [11]. Before the introduction of the new criterion in the legislation, there was little focus in Norway on structured assessment of patients` capacity to consent to treatment in mental health care. Following the amendment to the legislation, health care professionals have been given greater responsibility to assess a patient`s condition. They have to attend more closely to the patient's precise condition to be able to collaborate more fully with the patient and to make additional efforts for the patient to voluntarily engage in their care. Health care professionals need to look for signs and symptoms, and listen to the patient`s preferences to acquire knowledge of the patient’s condition in order to provide suitable treatment and care, and to adapt the care in the event of improvement or immediately take necessary steps in the event of deterioration. The term condition indicates a temporary state of illness or health, and provides information about a patient’s physical, mental and cognitive capacity at a specific point in time [12].

Community treatment orders (CTOs) have been introduced in most Western jurisdictions [13] with different options for intervening and treating patients under coercion [14]. Most CTOs stipulate that the patient must comply with what the health care provider considers to be necessary care and treatment, in order to avoid a relapse that requires re-admission to hospital [14–16]. The change to a model based on capacity to consent was considered particularly relevant in order to reduce the number of patients on CTOs. In discussions and consultations before the amendment, sceptics expressed concern about its consequences. They feared that patients with severe mental disorders and complex needs would avoid treatment, with serious implications for the health and welfare of patients and their families [17]. The Norwegian CTO scheme is summarized in Table 1.

**Table 1:** Norwegian CTO scheme

Norwegian CTO Scheme:
The CTO scheme was introduced in Norway with the Mental Health Care Act in 1961, and was continued based on an amendment to the Act in 2001. The scheme is based on clinical practice and each CTO is decided by the responsible psychiatrist or specialist psychologist. The conditions for implementing a CTO are the same as for involuntary inpatient treatment: patients must have a severe mental illness and either have an evident need for treatment or represent an imminent danger to their own life, or the life or health of others. A study of Norwegian CTOs has shown that they are solely based on patients` clear need for treatment (treatment criterion), with the addition in a few cases (18%) of the risk of posing a danger to themselves or others (harm criterion) [18]. In the case of involuntary medication treatment, a separate treatment decision is required. The legislation requires that coercion is considered necessary and a CTO presupposes that voluntary treatment has been unsuccessfully attempted, or it would be clearly futile to attempt this. Patients must also be offered adequate treatment and care that meet their needs. The CTO decision must be made on the basis of available information and a medical examination of the patient. An overall assessment must also be made as to whether a CTO is the best solution for the patient. In this assessment, patients must be allowed to express their opinion and particular emphasis must be placed on patients’ wishes, and how they feel about involuntary treatment. A CTO decision is valid for 12 months, but it must be re-assessed by the responsible professional at least every three months to determine whether the conditions are still met. If the CTO continues for more than 12 months, it must be approved by an independent review board (the Control Commission). In practice, this means that a patient may be under a CTO indefinitely. The CTO population in Norway has been shown to have the same patient characteristics as seen in studies from other jurisdictions [15, 18, 19]. There is no complete information on the numbers of CTOs in Norway, but in a study from 2012 that included a third of the population, the incidence rate was 23.8 and the prevalence rate was 47.4 per 100 000 inhabitants over the age of 18 [20].

The aim of this study is to explore patient experiences of how far the new legislation has enabled them to be involved in decisions on their treatment after they were assessed as capable of giving consent and had their CTO revoked due to the change in the legislation. The results are discussed in light of the intentions behind the new condition in the legislation.

## Method

### Design

The study used qualitative in-depth interviews to explore participants’ experiences and opinions. The interview and analysis processes were inspired by hermeneutics and a dialogical approach [21, 22]. The data were developed through dialogue between participants and researchers, where the researcher focuses on not seeking to confirm what she already knows, but instead attempts to be open to potential new understandings [21]. The study was conducted in specialist and primary health care in a region of Norway. The present article forms part of a larger study that examines the experiences of the new legislation of health care professionals, patients and their relatives.

### Involvement of service users

In order to design a study with a high degree of relevance and clinical benefit, four focus group interviews were conducted with distinct groups during the planning stage. The participants were 1) patients with experience of involuntary admission and CTO, 2) relatives of patients with experiences of involuntary admission and CTO, 3) health care professionals working in the community and 4) health care professionals working at a psychiatric hospital. Groups 3 and 4 both worked with patients who had experience of involuntary admission and CTO. The focus group interviews contributed to the development of the interview guides and gave the research team insight into what the various groups considered important to consider and explore in conducting the study.

At the start of the study, collaboration was established with a peer group of six people with personal experience of involuntary mental health care and CTOs as patients or relatives. This group of experts by experience contributed to discussions and made suggestions for the interview guide and the implementation of the interviews. This cooperation enhanced our understanding of what the amendment meant from their perspectives. A lived experience consultant was also engaged in the study.

### Recruitment

The participants were recruited from patients who had been on a CTO at the university hospital in the catchment area of the study. The inclusion criterion was patients who had their CTO revoked between 01.06.2017 and 01.09.2018, being assessed as competent to give consent. Fifty-five patients met the inclusion criterion during the study period. Random sampling was conducted among these patients. The last author had access to patient records to find out the patient’s age and the names of clinicians who had provided care to the patient. Those who knew the patient, but were not in charge of treatment, were contacted and given written and oral information to invite the patient to participate. Potential participants who no longer received care from the specialist health service were invited to participate by letter, followed by a telephone call from a lived experience consultant. Eighteen persons declined to be interviewed. When participants agreed orally to participate, the first author contacted them to clarify any questions and agree on the interview location. No participants withdrew during the study.

### Participants

The data consist of interviews with twelve participants aged 20–75 years, six women and six men. Four of them had a job, were studying or retired, and nine had disability benefits. Ten participants were single, two had partners and three had children. Two-thirds of them lived in urban areas and one-third in rural areas. Four participants rented or owned their homes, seven lived in supported council housing, while the accommodation of one was unknown. Participants had different levels of functioning. Several needed help with self-care, medication and practical tasks such as cooking and cleaning, while others only needed counselling. They received this support from the housing staff, mental health care staff or their doctor. One participant had regular voluntary hospital admissions and a few had treatment in an outpatient clinic. Eleven participants revealed their diagnosis, while one did not want to talk about his diagnosis. Nine had been diagnosed with schizophrenia spectrum disorders (ICD-10 F20-29), one with mood (affective) disorder (ICD-10 F30-39) and one within the category of disorders of adult personality and behaviour (ICD-10 F60-69). In addition, four were addicted to alcohol/drugs. One participant had been under a CTO once, seven had been under a CTO several times, while for one, the number of CTOs was unknown. The length of the CTOs had varied from three months to several decades. At the time of the interview, two of the participants were back on a new CTO.

### Interviews

Interviews were conducted by the first author from September 2019 to March 2020. Participants chose the location, and interviews took place in their homes or in the hospital. During the interviews, efforts were made to make participants feel comfortable and secure and to provide them with information suitable to their level of functioning.

The interviews lasted from 45–90 min; they were audio recorded and subsequently transcribed in their entirety and anonymized. Following each interview, field notes were written about the interview situation and the interviewer’s experience of the session.

The interview guide consisted of open questions and accompanying sub-questions based on the following topics: 1) Presenting oneself, one’s everyday life and one’s collaboration with health care professionals, 2) Experience of being under a CTO, and 3) Experiences of the change in the law and no longer being under a CTO. At the end of the interviews, the interviewer asked the participants about their experience of the interview situation.

### Analysis

A thematic analysis was conducted, with a hermeneutic approach inspired by Fleming et al. [21]. In hermeneutic analysis, researchers identify their horizon of understanding, understood as pre-understanding based on their background and experience and the context of the interviews and analysis. This approach presupposes critical reflection, dialogue and the capacity of researchers to see the significance of their own role in dialogue with participants and in interpretation of the data [21]. The authors have extensive experience of treatment and follow-up care of patients in involuntary mental health treatment and CTOs from their clinical work, counselling, advocacy or legal assistance. In a qualitative study with a hermeneutical approach, the researchers’ pre-understandings and experiences from the field are seen as a necessary basis for new understanding, although it is also vital to challenge one’s pre-understanding in order to understand in new ways. A movement back and forth between the whole and parts of the material, questions posed to the text and dialogue between the researchers are all necessary to achieve a new understanding [21].

The audio recordings were listened to and the transcripts read many times. Inspired by the analytical steps recommended by Fleming et al. [21], efforts initially concentrated on gaining an understanding of each interview as a whole, and as a context and condition for understanding the parts. In the next step, each sentence or passage was studied to grasp its meaning and enhance understanding. Particularly interesting statements or passages were highlighted in the text and questions, reflections and ideas were noted down in the margin and discussed by the researchers. The meaning units ranged from a few words to whole sentences and paragraphs, to ensure that the participants’ concepts were retained [23, 24]. The meaning units were discussed and preliminary topics were identified. We used the research question as a basis for deciding on the topics to continue with. Further rounds of reading were conducted. Questions were posed to the text about how to understand the various parts or statements, alternating with considering them in relation to the whole. The first understanding of the whole was challenged and revised by working on the parts. The movement from the parts back to the whole constituted the third step of the analysis. Themes changed and were continuously assessed in relation to participants’ statements, then retained or rejected, or new analytical concepts were identified. Quotes that represented the various themes were selected and sorted on large pieces of paper to gain an overall visual impression of themes and sub-themes. Some themes were interwoven and some new ones emerged. In order to understand the latent descriptions of participants’ experience, it was important that the first author had conducted the interviews. Through dialogue, the researchers challenged each other’s understandings based on their different backgrounds and experiences from the field, misunderstandings were eliminated and an effort was made to achieve a common understanding of the data. Dialogue with the text and relevant research literature helped to challenge the researchers’ pre-understandings and to develop the analysis. The analysis finally resulted in three main themes: 1) a feeling of greater autonomy and respect, 2) no change in condition and treatment, and 3) past experiences are not erased.

### Ethics

This study has been assessed by the Regional Ethics Committee (REK Nord) REK No. 2018/1659, and approved by the data protection officer of the University Hospital of North Norway.

In conducting this study, the researchers were aware that the participants’ mental health disorders could lead to changes in their condition and capacity to consent. During some interviews, it was necessary to assess participants’ understanding of what participation in research involved. To ensure that patients who were on CTOs at the time of the interview (*N* = 2) were able to make an autonomous assessment of their participation in the study, the clinicians treating them were asked to assess their capacity to consent to participation in the research.

All participants received oral and written information about the study, and were informed that participation was voluntary. They were also told that they could withdraw from the study at any time before the data had been included in the analysis, without giving a reason and with no negative consequences for them.

## Results

The analysis revealed three themes that show how the participants experienced having come off their CTO on the basis of capacity to consent.

### A feeling of greater autonomy and respect

Having their CTO revoked under the new legislation had a considerable impact on the participants. Several of them stated that coming off the CTO this time was a different experience from before. They experienced greater autonomy, freedom and respect. They also stated that it was the right decision to terminate the CTO, although they were very surprised because they did not feel that there was any change in their state of health. Several participants did not understand how it was possible to keep them in involuntary treatment for many years and then remove the coercive measures without any change in their condition. Some had received little information when the new legislation came into force and wondered to what extent changes in their level of functioning had played a part in the assessment of whether or not to continue the CTO.

Several participants found it difficult to make decisions about their own treatment after having been on a CTO for a long time. For some it was a great relief, while for others it was frightening. Klara was angry at first when her CTO was revoked because she was afraid of not receiving the same care and treatment without a CTO. It was difficult for her to understand how the change could have come so suddenly:

*“It all went so damn fast when the new law came, because I was used to being on a CTO all the time, then suddenly I was going to come off it. And then you think, well, bloody hell, now they’ve been giving me involuntary treatment for years and years, and then suddenly they want it to be voluntary… What was the point of having me on a CTO for so many years? And then suddenly, after the law was changed, did they change? … So, like, it doesn’t apply any longer?”*

After coming off the CTO, the participants were in a different position when collaborating with health care professionals. They found that the professionals were more likely to involve them in discussions and listen to their opinions, and they experienced respect. Several said that they participated more actively in collaboration; they offered their own opinion and were not afraid to disagree. No longer being under a CTO had a positive effect on their self-image, their dignity and their feeling of being more normal. Hans put it this way:

“I don’t want to talk about the way it was before. … Now people don’t think there’s something strange about me. …I don’t think they see anything wrong with me. …they respect me properly.”

The CTOs had been revoked over two years previously, and two of the participants were back in involuntary treatment again. Most participants said that they had not needed voluntary admissions to community mental health centres or hospitals. None had lost any treatment or care since their CTO was revoked, and they cooperated on treatment. Most participants believed that they would still be under a CTO if the legislation had not changed.

### No change in condition and treatment

Participants’ treatment and care had not changed as a result of the new legislation. The majority were offered and wanted to continue with the same care with some adjustments, e.g. their care provider changed from an outpatient clinic to primary health services. Some participants could still contact therapists at the community mental health centre or the regional psychiatric hospital as required, which was felt to be reassuring.

For most participants, their housing and everyday lives were unchanged. Those who previously lived in supported housing continued to live there. They were happy with the services offered, but wanted a more meaningful life with work and hobbies. Several stated that having had others decide things for them for many years had made them passive and lacking in initiative, and that they found it difficult to live meaningful lives. Several participants also had problems with irregular sleep patterns and with socializing. Many were distressed because of their disorder and medication; they felt lonely and found life monotonous.

A few participants wanted a different kind of accommodation because they found it challenging to have to constantly relate to staff and other residents with whom they had little in common. One participant felt that the staff focused too much on his illness and gave him too much advice about diet and cutting down on tobacco, alcohol and drugs. Pål, who was addicted to drugs and had considerable experience of involuntary treatment, said the following about the supported housing:

“It’s not obvious to me what are rules and what’s the involuntary stuff.”

Some participants found the regulations in council housing difficult to comply with. This was because their lives and disorders were often challenging enough in themselves, and because it seemed unreasonable or meaningless to have some of the rules in one’s own home. Problems with the regulations made one participant worry about being evicted and losing her right to council housing since the council no longer had the same responsibility since her CTO was revoked.

Participants who owned or rented their own home described a different structure to their lives, with work, education or various enjoyable activities. These participants also had severe mental disorders, but described improved mental health and greater independence to take control of their lives. Two who lived in their own homes felt that their life had greatly improved after coming off the CTO. They described enthusiastically how much it meant to regain their autonomy and have more freedom. They talked about reducing and adjusting their medication to make them feel better physically and have fewer side effects. Hedda said:

*“You get quite ... apathetic from taking medicines, they kind of dull your feelings. Now I’m taking Haldol. Haldol has a lot of side effects, maybe you can see the side effects in my mouth and eyes, they move a lot? … Haldol gives me such a chemical feeling in my body so it’s just awful! When I take it and it has a powerful effect on me. I used to take 12 milligrams, but now I’m on 3.5.”*

Hedda had never had a say in her medication for several years, but she described completely different cooperation after the CTO was revoked. Several other participants had similar experiences.

### Past experiences are not erased

In addition to having been on CTOs, all participants had experienced involuntary admission to hospital. They all described having been subject to various coercive measures, both during the process of being admitted and after admission. They talked about how it felt to be taken by force from their home for a compulsory examination, and to be forcibly medicated and restrained with belts. Their experiences of coercion also involved being prevented from making decisions on their own treatment, and being subject to various forms of compulsion over a long period of time. The participants’ stories of their experiences of coercion did not distinguish between a CTO and involuntary inpatient treatment. Their previous experiences of both inpatient and outpatient compulsory treatment were important factors in their current treatment, their sense of autonomy and their everyday life without coercion.

The participants’ many years of experience of various forms of coercion had made a lasting impression that affected their view of seeking help if their condition deteriorated. Although they now experienced greater trust and kindness among health care personnel, several had lost confidence in certain individual professionals. They felt vulnerable, being afraid of becoming acutely ill again and unsure whether they could be subject to coercion again if their condition worsened. Negative experiences of coercion made some participants afraid that it could happen again if there was a new crisis. Several participants had previously asked for help when their illness seemed to be deteriorating, but did not receive what they asked for. They received far more intrusive care than they requested and felt misunderstood or mistrusted. Anne explained:

*“I’d been to my doctor to get a sick note. And then I’d told him how I was feeling. For a long time I’d felt that someone was watching what I was doing. A few days later, a lady… who was a substitute for my GP… came to my house and said that I had to go to a psychiatric hospital. I couldn’t believe it was true! I thought she was joking! I was terrified!”*

Several participants mentioned how meetings with health care staff had been important to them, for better or for worse. Ole said that it made a difference whom he got to talk to when he rang the hospital, and added that it was not right that the treatment you are offered depends on which clinician you happen to talk to when you need help. Another participant, Pål, wanted to be inconspicuous and therefore mostly talked about everyday matters with the health care staff working in his supported accommodation. If they viewed him as psychotic, he was uncertain about the reactions and measures he could expect. He explained:

“I try to keep to my senses… otherwise I may get some unwelcome reactions.”

As they were unsure about the types of treatment and care offered, the participants found it difficult to tell clinicians about problems or experiences that could be interpreted as signs of illness; it could be difficult to be oneself and to ask for help at the same time. 

## Discussion

The aim of this study was to explore patient experiences of having come off a CTO due to their capacity to consent. The study shows that the participants experienced greater autonomy as a result of the new legislation. They also found that their care, treatment and accommodation remained largely unchanged. However, they were concerned that they could be subject to coercive measures again if their condition worsened.

The intention of the capacity-based mental health legislation in Norway was to achieve greater alignment between human rights and mental health care. In connection with the prevention and restriction of coercion and patient participation in health care decisions, mental health care services are now expected to cooperate with patients to find suitable solutions for treatment and care.

The capacity-based mental health legislation means that it is no longer possible to justify the use of coercion on patients needing maintenance treatment. This also applies to patients who have had successful medication treatment and thus regained their capacity to consent, but who are assumed likely to stop taking the medication when it becomes voluntary, leading to rapid deterioration. A feared consequence of the change in the legislation was that patients with severe mental disorders and complex needs would avoid treatment, with serious consequences for their health and welfare [17]. At the same time, it has previously been argued that patients under a CTO generally appear to have a level of functioning that indicates capacity to consent as long as they live and function outside an inpatient facility [25]. Our study shows that the participants still wanted care and treatment after their CTO was revoked. Almost all the participants had collaborated on voluntary medication and follow-up care for over two years following the termination of their CTO. Only two participants had needed involuntary admission or a new CTO, two years after their CTO was revoked.

This contrasts with the participants’ previous experience of periods when they were not on a CTO. When CTOs were revoked previously, they were not listened to or consulted regarding their treatment and care even though this was voluntary. This suggests that being considered as having capacity to consent meant that participants were now more valued and respected, with a new status and position with regard to their treatment and collaboration with health care personnel, involving dialogue and more information. Increased self-determination as described by the participants is in line with the aim of the amended legislation [9]. Several participants pointed out that they still found it difficult to relate to their housing regulations and advice from staff on diet and abuse of alcohol or drugs. Supported housing can provide security and protection, but can also be perceived as invasive or overprotective, which affects quality of life and whether the housing feels like a home [26].

The amendment to the legislation stipulates that a systematic assessment of the patient’s condition must be performed by a professional responsible for the patient. All study participants had a severe mental disorder, which meant that they were dependent on everyday help to varying degrees. The nature of such disorders often includes periods of deterioration which may reduce patients’ ability to assess their own situation and to make decisions [27]. The shift from a diagnostic focus to a focus on functioning means that changes in patients’ condition must be taken into account to a greater extent. It has been argued that this calls for changes in health care professionals’ attitudes and their views on which patients need to be subject to involuntary treatment [28]. The results from our study show that patients experienced a change in how health care personnel interacted with them after the change in the law, being more often included in dialogue and decision-making. This indicates that the new legislation has opened a new window of opportunity and new forms of cooperation in the treatment of severe mental disorders.

The amendment to the Norwegian Mental Health Care Act provides assessment guidelines for those responsible for decisions on the use of coercion. A Norwegian supreme court ruling from 2018 regarding a patient discharged from a CTO raised the question of what was required for lack of capacity to consent to be a condition for the use of coercion [29]. The ruling confirms that the decisive factor must be the extent to which the illness affects patients’ ability to make realistic assessments of their condition and the consequences of treatment decisions. The ruling states: “*Patients with a fair degree of realistic insight into their own situation should be able to decide for themselves whether they want to receive health care. This also applies when there is a question of whether long-term medication has given them back the ability to understand. Unlike in the past, they can now decide to end their treatment even if health care personnel think this is unfortunate*” [29]. This demonstrates legal practice that follows the intention of the legislation, i.e. the patient’s right to self-determination shall be decisive as long as the capacity to consent is present.

The majority of the participants in our study were living stable lives with a disorder that was manageable at the time of the interview. Nevertheless, several were afraid that their condition could deteriorate, leading to loss of control and involuntary treatment again. This fear was quite marked in a number of participants, but seemed to be less so in those who had trusting relationships with health care professionals. A trusting relationship is key to patient-clinician collaboration, but is often challenged when treatment is involuntary. Several of the participants in this study described trusting relationships with health care professionals despite the power imbalance in a CTO. This is also emphasized in a study that finds that trusting relationships can be achieved by health care professionals who show confidence in patients, are seen as sincerely concerned about their best interests, and are honest, reliable and good listeners [30]. It is also important that professionals have sufficient knowledge to interpret early signs of a negative development in the disorder to enable them to provide the necessary treatment and care to avoid loss of control and acute admission to hospital. This is clearly vital to maintain patient autonomy [27]. To understand the nature of a disorder, it is not sufficient to know how the patient is feeling, but also how the disorder, e.g. psychosis, may develop [31]. This requires knowledge of how illnesses and disorders arise and how to proactively anticipate a flare-up to prevent exacerbation and the development of severe illness [32]. Also important here are good insight and the capacity to understand how patients experience their illness and what is helpful.

Previous studies have shown that patients on CTOs in Norway felt that their mental health care was a far-reaching intrusion in their lives that hindered their self-expression [33, 34]. Efforts to increase participation of seriously ill mental health patients in their treatment and care have been proceeding for many years. However, it was not until the introduction of the condition of lack of capacity to consent that mental health patients with such capacity became legally entitled to refuse treatment they did not want in the same way as patients in physical health care. The amendment to the Mental Health Care Act may thus represent part of an ongoing paradigm shift in the treatment and care of seriously ill mental health patients with complex needs in Norway.

## Strengths and limitations

This study focuses on patients’ perspectives and experiences, and aims to provide first-hand knowledge of the experience of having come off a CTO based on capacity to consent. Therefore, the study has not included any other perspectives on the change in the legislation, such as those of health care personnel and patients’ relatives. The changes and experiences described by participants may have been influenced by various factors in their lives, and cannot be traced back to the amendment alone. Some participants reported not having received information about the change, but it may be said to strengthen the results that these patients also experienced a marked improvement in their autonomy and involvement in their treatment and care.

The participants were recruited from lists of patients in the catchment area whose CTO had been revoked during the study period, based on strategic sampling. Treatment personnel have thus had no influence on recruitment. In this way, we have aimed at a varied sample of participants. The study had a small number of participants, while a larger number would have been able to provide a greater variety of opinions and experiences.

The project group collaborated with a peer group of people with experience of CTOs as patients or family members. This collaboration gave the research team valuable insights into conducting recruitment and interviews. The original plan was to include the peer group in the data analysis, but this was not feasible due to the COVID-19 pandemic.

## Conclusion

For patients in this study with previous experience of a CTO, it would seem that the changed legislation has worked as intended. The study shows that health care is largely provided in accordance with the patient’s wishes. A systematic assessment of capacity to consent seems to lead to a greater emphasis on patients’ opinions, state of health and level of functioning in their treatment and care. The participants experienced improved dialogue, information and assistance in collaboration with health care professionals. This helped them to make more informed choices and to be more actively involved in decisions on their treatment. The change in the legislation may indicate that new forms of patient-clinician collaboration are emerging in mental health care, where patients are trusted and their views taken seriously. The participants experienced a notable reduction in both formal and informal coercion.

As a step in improving treatment and care for people with severe mental illness and reducing the use of coercion, there is a need for studies that include the perspectives of health care professionals and patients’ relatives. Knowledge is needed on how relatives experience the new situation, and on whether their role and responsibilities have changed since their family member came off the CTO and gained more autonomy. A further area for exploration is health care professionals’ experiences of providing care and treatment with and without a CTO, and how far they feel they should accommodate patient wishes.

## Data Availability

In order to protect the anonymity of the participants, the data on which this manuscript is based will not be made generally available, with the exception of the data that has been carefully selected for presentation in the manuscript.

## Abbreviations

CRPD: The Convention on the Rights of Persons with Disabilities
CTO: Community treatment order
